# *In-situ* Electric Field-Induced Modulation of Photoluminescence in Pr-doped Ba_0.85_Ca_0.15_Ti_0.90_Zr_0.10_O_3_ Lead-Free Ceramics

**DOI:** 10.1038/srep28677

**Published:** 2016-06-24

**Authors:** Hai Ling Sun, Xiao Wu, Tat Hang Chung, K. W. Kwok

**Affiliations:** 1 Department of Applied Physics, The Hong Kong Polytechnic University, Kowloon, Hong Kong, China

## Abstract

Luminescent materials with dynamic photoluminescence activity have aroused special interest because of their potential widespread applications. One proposed approach of directly and reversibly modulating the photoluminescence emissions is by means of introducing an external electric field in an *in-situ* and real-time way, which has only been focused on thin films. In this work, we demonstrate that real-time electric field-induced photoluminescence modulation can be realized in a bulk Ba_0.85_Ca_0.15_Ti_0.90_Zr_0.10_O_3_ ferroelectric ceramic doped with 0.2 mol% Pr^3+^, owing to its remarkable polarization reversal and phase evolution near the morphotropic phase boundary. Along with *in-situ* X-ray diffraction analysis, our results reveal that an applied electric field induces not only typical polarization switching and minor crystal deformation, but also tetragonal-to-rhombohedral phase transformation of the ceramic. The electric field-induced phase transformation is irreversible and engenders dominant effect on photoluminescence emissions as a result of an increase in structural symmetry. After it is completed in a few cycles of electric field, the photoluminescence emissions become governed mainly by the polarization switching, and thus vary reversibly with the modulating electric field. Our results open a promising avenue towards the realization of bulk ceramic-based tunable photoluminescence activity with high repeatability, flexible controllability, and environmental-friendly chemical process.

Rapid and reversible manipulations of photoluminescence (PL) activity in luminescent materials have attracted much attention due to wide applications, including long-distance quantum communication, photonic devices, volumetric 3D display, back light, and biomedicine^1^,^2^,^3^,^4^,^5^,^6^,^7^. Until now, the modulation of PL is commonly achieved by adjusting the composition of host materials and/or doping various rare-earth ions (RE^3+^) via chemical approach in phosphor^5^,^8^,^9^,^10^. However, these approaches are irreversible and not favorable for practical applications. They also provide no opportunities to elaborate the mutative PL process, which is essential for exploring the underlying physical mechanism. In this regard, an *in-situ* and real-time approach for PL modulation has been proposed by applying an external electric field on specific host materials such as organic thin films^11^,^12^ and ferroelectric titanate films^2^,^13^,^14^. As known, ferroelectrics are materials that have spontaneous electric polarization (or electric dipoles) that can be reversed by the application of an external electric field.

An important assumption underlying much of the electric field modulation work is that the reversible variation of PL should be attributed to electrically changing the structural symmetry of the host and thus tailoring the local crystal field around the RE^3+ ^15,16,17. Low-symmetry hosts typically exert a crystal field containing more uneven components around the dopant ions compared with high-symmetry counterparts^16^,^18^. The uneven components enhance the electronic coupling between 4f energy levels and higher 4f5d configuration and subsequently increase f–f transition probabilities of the dopant ions^19^. The common approaches for ferroelectric hosts to rationally tune structural symmetry are based on those coupling physical factors that can respond well to external electric field such as biaxial strain^14^,^20^, polarization^2^,^21^,^22^,^23^, or phase transition^24^,^25^.

Despite a significant modulation of PL by applying external electric field has been realized in a reversible and *in-situ* manner, this achievement is only reported for ferroelectric thin films^2^,^13^,^14^. It is known that bulk ceramics offer several advantages compared with thin films, including higher repeatability, much better chemically inert and thermal stability, superior mechanical properties, easier preparation process and lower cost to meet the market requirements. Among the environmental-friendly ferroelectric ceramics, Ba_0.85_Ca_0.15_Ti_0.90_Zr_0.10_O_3_ (abbreviated as BCTZ) possesses excellent piezoelectricity owing to its composition proximity of the morphotropic phase boundary (MPB) to a tri-critical triple point^26^. Inspired by its remarkable polarization reversal and phase revolution around room temperature, BCTZ is chosen as the ferroelectric host in this work for providing a considerable change in structural symmetry under electric field. In addition, these features also provide the ceramic a highly reliable response to electric field change and allow the use of a low voltage for inducing the structural change.

Meanwhile, Pr-doped luminescent materials have shown great potential in applications, such as red phosphor for the display applications^27^,^28^, sensors and optical-and-electro multifunctional integration^29^,^30^,^31^. Unlike other RE^3+^, Pr^3+^ has a unique characteristic. Namely, resulting from the close energy separation between the ^1^S_0_ level and the lowest edge of the 4f5d configuration, its emission depends strongly on the position of the 4f5d configuration, which is extremely influenced by the characteristics of the host^32^,^33^,^34^. For instance, the PL performances of Pr-doped ferroelectric ceramics can be enhanced greatly by electric field, as a result of the lowering of structural symmetry in the host after poling^22^,^35^. Owing to the high sensitivity, the emissions of Pr^3+^ have also been utilized as a structural transition probe for investigating phase transition of ferroelectric hosts that induces crystal-symmetry changes^36^,^37^,^38^. On the other hand, the ferroelectric and piezoelectric properties of the host materials can also be improved by the doping of Pr^29^,^39^.

In this work, we report the real-time PL modulation in bulk Ba_0.85_Ca_0.15_Ti_0.90_Zr_0.10_O_3_ ferroelectric ceramic doped with 0.2 mol% Pr^3+^ (abbreviated as BCTZ:Pr). To the best of our knowledge, there is no report on direct PL *in-situ* modulation of bulk ceramics showing a reversible manner by applying external electric field. For BCTZ, its structural transition between tetragonal and rhombohedral phases as well as the dipole switching is considerably susceptible of electric field due to the MPB effect. Accordingly, we aim to modify the structural symmetry of the BCTZ host with a dynamic electric field, thus tailoring the local crystal field around Pr^3+^. Figure 1 shows the schematic of a sandwiched structure used for continuously detecting the down-shifting PL emissions of BCTZ:Pr. A silver electrode (~10 μm) was first coated on the back surface of a thin ceramic disk with a thickness of ~200 μm, and then a conductively transparent electrode of indium tin oxide (ITO) (~0.4 μm) was deposited on the top surface at 250 °C by magnetron sputtering. The transparent ITO enables the excitation and emission light to pass through under an external electric field applied along the thickness direction (Fig. 1a). With switching on the external electric field, changes in PL are induced via modulating phase transition and polarization switching (Fig. 1b,c) by virtue of the extremely high sensitivity of the local crystal field around Pr^3+^ to external electric field^2^,^20^.

## Results and Discussion

### *In-situ* Structure and Phase Transition

**Figure 2:**
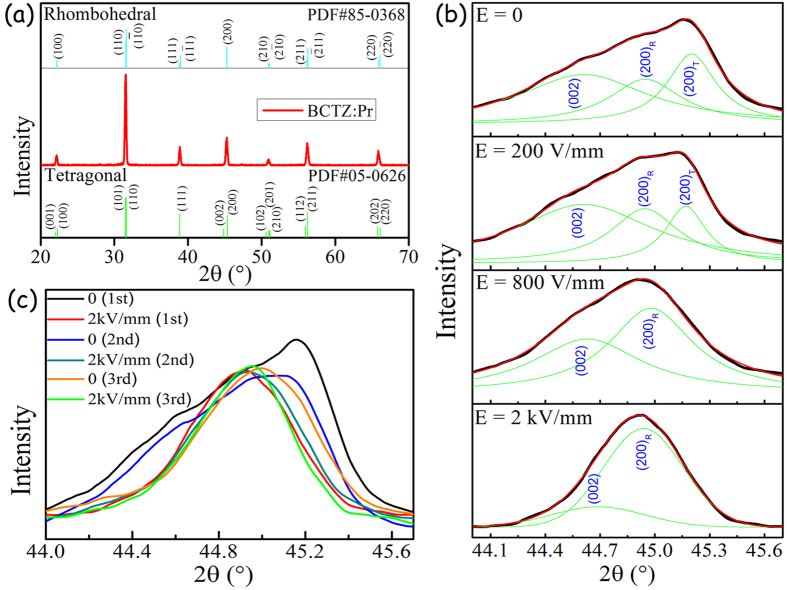
XRD pattern of the BCTZ:Pr ceramic. (**a**) General scan in the range of 20 to 70°. (**b**) Fine scan under an external dc electric field of 0, 0.2, 0.8 and 2 kV/mm, respectively. (**c**) Fine scan with applying the off- and on-fields for three times.

The X-ray diffraction (XRD) pattern of the BCTZ:Pr ceramic is shown in Fig. 2a, revealing a pure perovskite structure of the ceramic. No additional peaks reflecting the presence of rare-earth oxides are observed, which suggests that the Pr^3+^ have diffused into the BCTZ lattice. The standard diffraction peaks are cited from the tetragonal (T) BaTiO_3_ (PDF#05-0626) and rhombohedral (R) BaTiO_3_ (PDF#85-0368), indicated by the vertical lines for comparison (Fig. 2a). Regarding the fact that the BCTZ ceramic derives from MPB with the coexistence of tetragonal (P4mm) and rhombohedral phases (R3m) around room temperature^26^,^40^, there is no visible evidence of the splitting of (200) peak at 2θ~45° (Fig. 2a). A broad diffraction peak is observed around 44°–46° as shown in Fig. 2b. It should be noted that the enlarged XRD pattern shown in Fig. 2b was obtained by fine X-ray scanning on the ceramic ready for applying an *in-situ* electric field, i.e., with both Ag and ITO electrodes. Owing to the complexity (coexistence of phases) and interference arisen from the strong diffraction peaks of ITO, the Rietveld refinement method has not been used for precisely identifying the crystal structure. Instead, with the primary aim of verifying the change of crystal structure, the broad diffraction peak has been resolved by the practical peak fitting method^25^, giving the fitting results indicated in Fig. 2b. For the ceramic without an electric field (E = 0), it is characterized by the obvious (200)^T^ and (200)^R^ peaks, which indicates that the coexistence of tetragonal and rhombohedral phases (even including an intermediate orthorhombic sub-phase (002)) at room temperature^40^. Under an electric field of 0.2 kV/mm, the (200)^T^ peak shrinks and becomes similar in size to the (200)^R^ peak (Fig. 2b). As the *in-situ* electric field increases to 0.8 kV/mm, the tetragonal (200)^T^ peak almost disappears whereas the (200)^R^ peak grows to the dominant one. Finally, the diffraction peak becomes relatively narrow with an evident (200)^R^ peak under an electric field of 2 kV/mm, revealing a transformation from a mainly tetragonal phase into a mainly rhombohedral phase. This unique ferroelectric phase evolution corresponds to the symmetry-raising characteristic, and agrees well with previous literature^21^ and the established ferroelectric phase diagram^26^. Although the (002) peak cited by the orthorhombic phase may exist in the ceramic, we regard it as an instable intermediate phase induced by E-field dependent phase evolution^41^,^42^.

For studying the reversibility of the phase transformation, the electric field has been switched off and on between 0 and 2 kV/mm for a number of times, and the XRD patterns *in-situ* with the off- and on-fields for another as-prepared sample have been measured, giving the results shown in Fig. 2c. The transformation under the first off- and on-fields has been discussed above and the XRD patterns are similar to those shown in Fig. 2b. As shown in Fig. 2c, the diffraction peak becomes broadened again when the field is switched off (i.e., 2^nd^). However, the peak does not restore to the original shape (i.e., 1^st^), in particular for the (200)^T^ peak, suggesting that the tetragonal phase cannot be completely recovered. Furthermore, the (200)^T^ peak is even hardly observed in the subsequent switching processes (i.e., 3^rd^). Instead, only the (200)^R^ peaks is clearly observed at the same diffraction angle 2θ~44.9° under both the on- and off-fields. These suggest that the field-induced ferroelectric phase transition takes place indeed but is not completely reversible. It takes at least three cycles of electric field to completely irreversibly transform the phase from tetragonal to rhombohedral, which then becomes unaffected by the electric field.

### Electrical Properties

**Figure 3:**
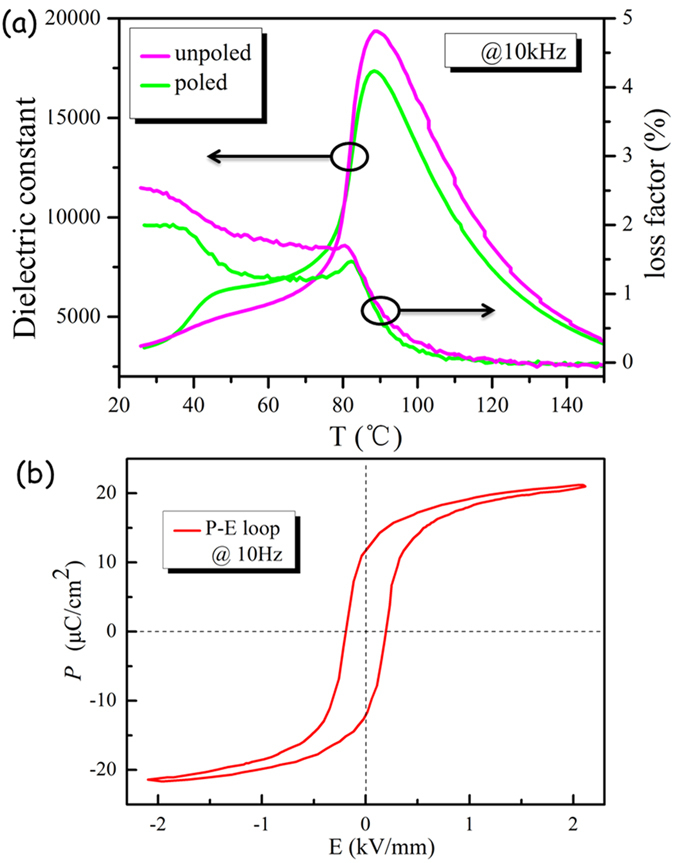
(**a**) Temperature dependences of ε_r_ and tan*δ* at 10 kHz for the un-poled and poled BCTZ:Pr ceramic, (**b**) *P-E* loop of the BCTZ:Pr ceramic at 10 Hz.

The temperature dependences of dielectric constant (ε_r_) and dielectric loss factor (tan*δ*) at 10 kHz for the un-poled and poled BCTZ:Pr ceramics are shown in Fig. 3a. For the un-poled sample, a strong peak corresponding to the tetragonal-cubic phase transition (T_C_~85 °C) is clearly observed in the temperature range of 25–150 °C. As evidenced by the variation of tanδ, a very weak peak assigned to the rhombohedral–tetragonal phase transition (T_R-T_) is barely observed around 40 °C. After poling under a dc electric field of 2 kV/mm, the weak transition peak (in ε_r_) becomes stronger and can easily be observed at similar T_R-T_. This should be arisen from the increase in rhombohedral phase, and thus provides evidences for the irreversible ferroelectric phase evolution induced by the poling field at room temperature (Fig. 2c). Figure 3b plots the ferroelectric hysteresis loop (*P-E* loop) of the BCTZ:Pr ceramic. As seen, it displays a typically saturated shape, and the remanent polarization P_r_ and coercive field E_c_ are about 11.8 μC/cm^2^ and 0.21 kV/mm, respectively. In addition, the ceramic also exhibits good electrical properties, such as a high piezoelectric coefficient *d*_33_ (~410 pC/N), large electromechanical coupling factor *k*_p_ (~50.6%), high *ε*_r_ (~3620) and low tanδ (~1.6%).

### Photoluminescence Excitation and Emission

**Figure 4:**
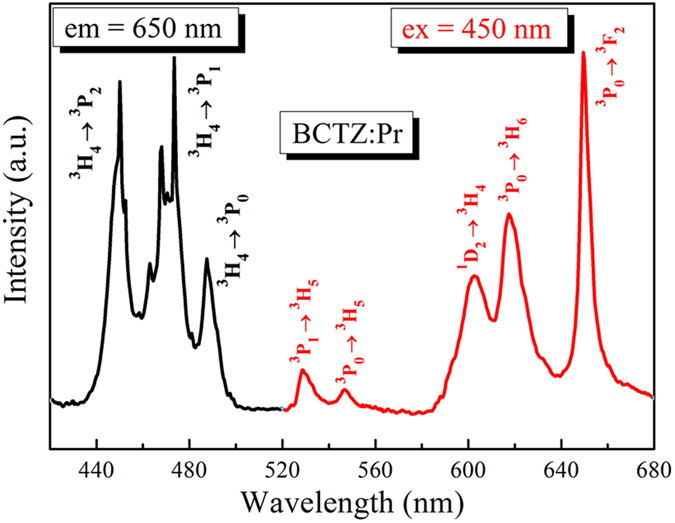
(**a**) Photoluminescence excitation spectrum of the BCTZ:Pr ceramic. (**b**) Photoluminescence emission spectrum of the BCTZ:Pr ceramic.

The photoluminescence excitation (PLE) spectrum monitoring the typical red emission of Pr^3+^ (650 nm) of the BCTZ:Pr ceramic is shown in Fig. 4. Three strong excitation peaks arising from the excitations of Pr^3+^ from the ^3^H_4_ ground level to the ^3^P_2_, ^3^P_1_ and ^3^P_0_ excited levels are observed at 450, 473 and 487 nm, respectively^18^,^39^. Accordingly, the photoluminescence (PL) emission spectrum of the ceramic has been measured under an excitation of 450 nm, giving the results shown in Fig. 4. Similar to other Pr-doped luminescent materials^23^,^34^, the ceramic exhibits strong red (650, 617, 602 nm) and weak green (547, 529 nm) emissions, which are attributed to the transitions (^3^P_0_ → ^3^F_2_, ^3^P_0_ → ^3^H_6_, ^1^D_2_ → ^3^H_4_), and (^3^P_0_ → ^3^H_5_, ^3^P_1_ → ^3^H_5_), respectively. Probably due to the slight differences in compositions which could influence the cross-relaxation process of Pr^3+ ^43, the strongest red emission occurs at 650 nm (Fig. 4), instead of 600 nm reported for the (Ba_0.85_Ca_0.15_)_1-x_Pr_x_(Zr_0.1_Ti_0.9_)O_3_ ceramics^21^. Owing to its strong intensity and f-f transition characteristics, the emission at 650 nm has been selected for studying the dynamic PL emissions under external electric field.

### *In-situ* PL Modification

**Figure 5:**
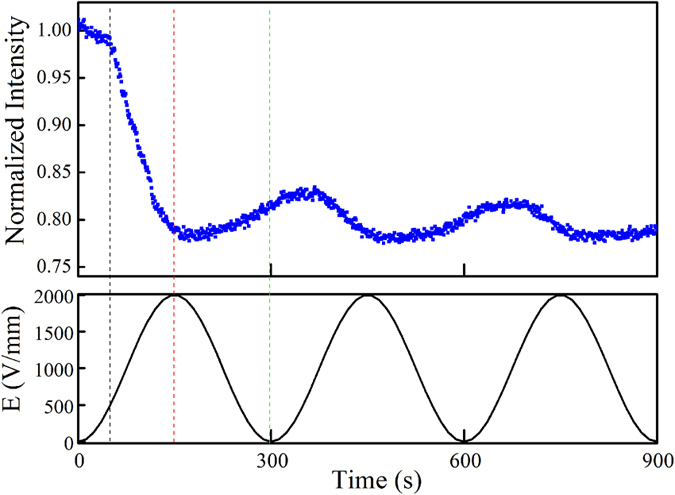
Dynamic photoluminescence responses of the BCTZ:Pr ceramic for the 1^st^ to 3^rd^ cycles of electric field.

Figure 5 shows the dynamic PL responses of the BCTZ:Pr ceramic under a biased ac electric field E which varies sinusoidally between 0 and 2 kV/mm. It is of great interest to notice that the observed PL intensity varies in a similar manner of E, suggesting that the PL emissions could be modulated reversibly, to some extent, by an external electric field. Indeed, different mechanisms may be involved in modulating the PL emissions, in particular for the first half cycle of E. As shown in Fig. 5, the observed PL decreases first slowly as E increases, and then falls rapidly at E > 0.5 kV/mm. The dramatic PL quenching (~22%) at high E should be attributed to the field-induced phase transformation as evidenced by the *in-situ* XRD analysis illustrated in Fig. 2. It has been known that the PL emissions are affected by the local crystal field around RE^3+^ and thus the structural symmetry of the host in accordance to the Judd-Ofelt (J-O) theory^44^,^45^; namely, a high structural symmetry will lead to a weak PL emission. For the BCTZ:Pr ceramic, it possesses a mainly tetragonal phase and then lower structural symmetry at low electric fields (Fig. 2b). As E increases to 2 kV/mm, it undergoes a transformation to a mainly rhombohedral phase, and thus engendering an increase in structural symmetry, which consequently leads to a decrease in PL intensity. As E decreases from 2 kV/mm to zero (i.e., the other half cycle of E), the observed PL intensity increases but cannot restore to the original value. There is a drop of 18% in PL intensity after the first cycle of E. This should also be attributed to the field-induced phase transformation which has been shown only partially reversible^46^. As evidenced in Fig. 2c, the ceramic contains more rhombohedral phase after the first cycle of E, and hence possesses higher structural symmetry and exhibits weaker PL emissions.

It should be noted that polarization switching also occurs in the first half cycle of E, which, however, enhances PL emissions as a result of the lowering of structural symmetry and the resulting increase in uneven components of the local crystal field around Pr^3+^. The uneven crystal field can mix opposite-parity states into the 4f configuration level and then increase the 4f-4f electric dipole transition probability of Pr^3+ ^16,18. Apparently, the polarization switching and phase transformation have opposite effects on PL emissions, and, on the basis of the results (Fig. 5), the effect of the latter is dominant. As illustrated in Fig. 3b, most of the ferroelectric dipoles are switched for alignment by an electric field smaller than 0.6 kV/mm, suggesting the resulting enhancement in PL emissions should become saturated at similar electric field. Probably due to the limited counter-effect, the observed PL intensity (which is modulated mainly by phase transformation) starts to decrease rapidly at E~0.5 kV/mm as shown in Fig. 5. Also, as illustrated in Fig. 3b, part of the aligned ferroelectric dipoles (about 40%) will relax as the electric field decreases from 2 kV/mm to 0, which consequently increases the structural symmetry and then weakens the PL emissions.

**Figure 6:**
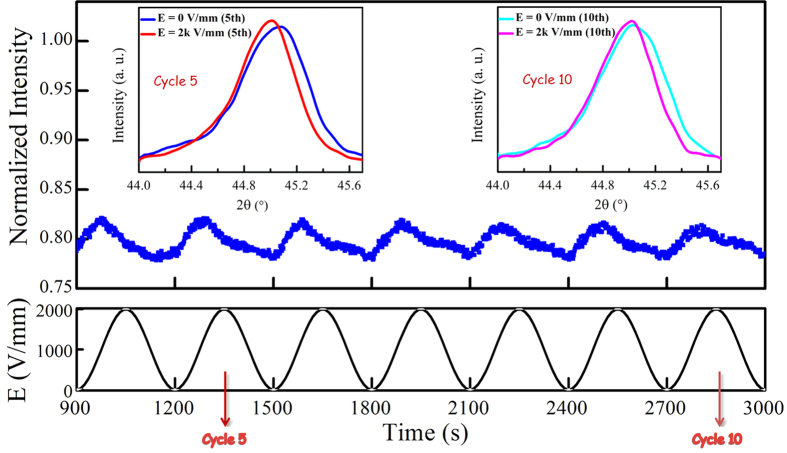
Reversible photoluminescence responses of the BCTZ:Pr ceramic for the 4^th^ to 10^th^ cycles of electric field. The insets show the XRD patterns of the ceramic under the off- and on-fields in the 5^th^ and 10^th^ cycle of electric field, respectively.

As evidenced in Fig. 2c, it takes at least three cycles of electric field to complete the irreversible tetragonal-to-rhombohedral transformation. Consequently, the effect of phase transformation on PL emissions decreases continuously in the 2^nd^ and 3^rd^ cycles of E, and becomes vanished in the subsequent cycles. The observed PL intensity is then modulated mainly by polarization switching, and thus starts to increase, instead of decrease, as E increase from 0 in each subsequent cycle of E, in particular for the 4^th^ to 10^th^ cycles as shown in Fig. 6. Although no phase transformation is induced, the applied electric field may induce local crystal strain, and thus deforming the rhombohedral structure slightly as indicated by the XRD results shown in the insets of Fig. 6. The change is reversible and leads to an increase in structural symmetry^14^,^47^ and then a decrease in PL intensity. However, it is very weak as compared with that arisen from polarization switching which increases the PL emission as discussed above. As a result, the observed PL intensity follows mainly the variation of E, but starts to decrease before E reaching its amplitude in each cycle (Fig. 6).

## Conclusion

In summary, we report a real-time modulation of down-shifting PL emissions of the BCTZ:Pr bulk ceramic via the application of an external electric field. The remarkable polarization reversal and phase evolution of BCTZ:Pr deriving from a tri-critical point near MPB enable reliable responses to the applied electric field. As evidenced by *in-situ* X-ray diffraction analysis, the applied electric field induces not only typical polarization switching and minor crystal deformation, but also tetragonal-to-rhombohedral phase transformation of the host. Unlike the former two responses, the field-induced phase transformation is irreversible, but engenders greater effect on PL emissions as a result of an increase in structural symmetry. After the phase transition is completed in a few cycles of electric field, the PL emissions become governed mainly by the reversible polarization switching. Owing to the minor and opposite effect arisen from the field-induced local crystal strain, the observed PL intensity however deviates slightly from the modulating electric field. Herein, this work could pave the way for PL modulation of bulk ceramic-based device applications, such as further electric controlled photoluminescence down-convertors.

## Methods

### Samples

The raw materials used to fabricate the Ba_0.85_Ca_0.15_Ti_0.90_Zr_0.10_O_3_ ceramics with 0.2 mol% Pr doping were commercially available carbonate powders and metal oxides: BaCO_3_ (99.5%), CaCO_3_ (99%), TiO_2_ (99.9%), ZrO_2_ (99.9%) and Pr_6_O_11_ (99.9%). The powders in the stoichiometric ratio were ball-milled thoroughly in anhydrous ethanol using zirconia balls for 12 h, then calcined at 1200 °C for 2 h in air before ball-milled again for 12 h and sieved through an 80-mesh screen. After mixed thoroughly with a PVA binder solution, the resulting mixture was pressed into disk samples. Then, the samples were finally sintered at 1350 °C for 2 h in air for densification. The ceramics were poled under an electric field of 2 kV/mm at room temperature in silicone oil for 30 min. Before the measurements of *in-situ* XRD and PL properties, a silver electrode was first fired on one of the surfaces while an indium tin oxide (ITO) transparent electrode was deposited on the other surface by sputtering technique.

### Measurements

The crystallite structure was examined using XRD analysis with Cu*K*_α_ radiation (SmartLab, Rigaku Co., Japan). *ɛ*_r_ and tan *δ* were measured as functions of temperature using an impedance analyzer (HP 4194A, Agilent Technologies Inc., Palo Alto, CA). A conventional Sawyer-Tower circuit was used to measure the *P-E* loop at 10 Hz. The piezoelectric coefficient (*d*_33_) was measured using a piezo-*d*_33_ meter (ZJ-3A, China).

The DC PLE and PL visible emission spectra were measured by a spectrophotometer (FLSP920, Edinburgh Instruments, UK) using a 450-nm xenon arc lamp (Xe900) as the excitation source. A voltage source (2410 1100V Source meter, Keithley Instruments Inc., US) was used to apply external voltage for investigating the E-field dependent XRD and PL properties.

## Additional Information

**How to cite this article**: Sun, H. L. *et al*. *In-situ* Electric Field-Induced Modulation of Photoluminescence in Pr-doped Ba_0.85_Ca_0.15_Ti_0.90_Zr_0.10_O_3_ Lead-Free Ceramics. *Sci. Rep.* **6**, 28677; doi: 10.1038/srep28677 (2016).

## Figures and Tables

**Figure 1:**
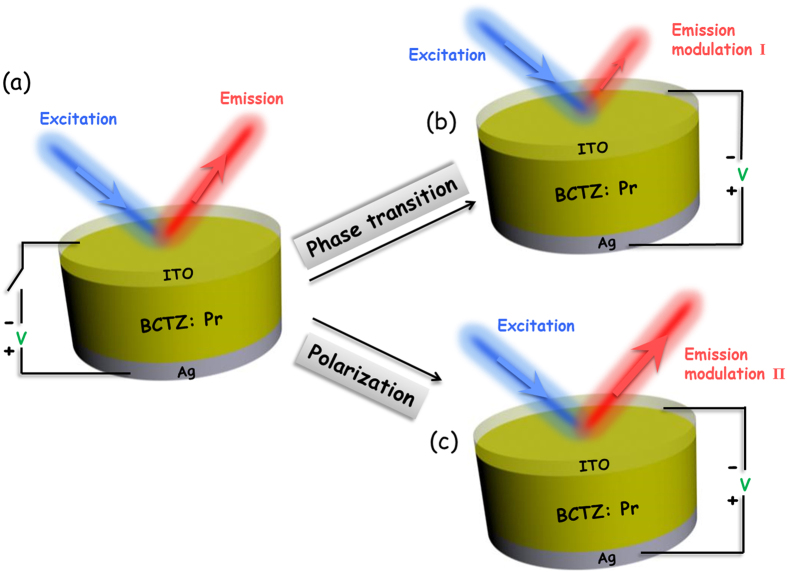
Schematic illustration for continuously detecting the real-time down-shifting photoluminescence emissions of the BCTZ:Pr ceramic when an external electric field is (**a**) switched off, and (**b,c** simultaneously) switched on.
